# Revealing the Nanoparticle-Protein Corona with a Solid-State Nanopore

**DOI:** 10.3390/ma12213524

**Published:** 2019-10-28

**Authors:** Diego Coglitore, Pierre Eugene Coulon, Jean-Marc Janot, Sébastien Balme

**Affiliations:** 1 Institut Européen des Membranes, UMR5635, Université de Montpellier CNRS ENSCM, Place Eugène Bataillon, 34090 Montpellier, France; diego.coglitore@gmail.com (D.C.); jmjanot@univ-montp2.fr (J.-M.J.); 2 Laboratoire des Solides Irradiés, École polytechnique, Université Paris-Saclay, Route de Saclay, 91128 Palaiseau CEDEX, France; pierre-eugene.coulon@polytechnique.edu

**Keywords:** protein corona, nanopore, single molecule

## Abstract

Protein adsorption at the liquid–solid interface is an old but not totally solved topic. One challenge is to find an easy way to characterize the protein behavior on nanoparticles and make a correlation with its intrinsic properties. This work aims to investigate protein adsorption on gold nanoparticles and the colloidal properties. The protein panel was chosen from different structural categories (mainly-α, mainly-β or mix-αβ). The result shows that the colloidal stability with salt addition does not depend on the structural category. Conversely, using the single nanopore technique, we show that the mainly-α proteins form a smaller corona than the mainly-β proteins. We assign these observations to the lower internal energy of α-helices, making them more prone to form a homogeneous corona layer.

## 1. Introduction

The interactions between proteins and nanoparticles were widely studied in the last decades, and from this emerged numerous open questions and applications in different research areas [1,2,3]. For instance, understanding the relationship between the structure and activity of a protein adsorbed on nanoparticles is essential to designing catalyst materials or sensors [4,5,6]. Gold nanoparticles (Au-NP) are the most intriguing, due to their ability to easily form a shell composed of one or more layers of proteins [7,8,9,10]. Depending on the kind of interaction, it can be named a “hard” or “soft” corona [11,12]. The protein corona can be characterized by different techniques. Circular dichroism gives information about the protein structure. Measurement of the corona size can be achieved by dynamic light scattering (DSL) or fluorescence correlation spectroscopy (FSC). FCS is more sensitive to small particles than DLS, but it requires protein labelling with fluorescent dye. On the other hand, DLS is a label-free technique, but information on single molecules/particles can be difficult to extract when aggregates are present in the mixture.

Single nanopore technology is one of the label-free single-molecule detection methods that emerged in the last three decades [13,14]. Besides the biological nanopore, which is the most efficient method to sequence [15,16,17] and identify biomacromolecules [18,19,20], the solid-state nanopore is the most suitable to detect folded proteins [21,22], aggregates [23,24,25,26,27] or nanoparticles [28,29,30]. They permit characterizing their size, shape or charge using more and less complex models [31]. Indeed, nanopores decorated with a lipid bilayer were previously reported for protein 5D fingerprinting sensing [32]. In this work, an elegant model to deduct the protein volume from its form factor is proposed. This model was recently improved to take into account the nanoparticle and nanopore surface charge and applied to the detection of DNA-coated polystyrene microspheres [28,29].

Since the nanopore approach has been demonstrated to be suitable to detect nanoparticles, also giving an estimation of the volume, we have recently used it to investigate the BSA adsorption on gold nanoparticles [33]. We have reported that the impact of resveratrol has a strong influence on BSA/AuNP interactions. Following this work, we now use a solid-state nanopore in order to characterize the first corona layer of proteins adsorbed on AuNP. We aim to demonstrate that nanopores can be efficient to determine the volume of the protein corona even if AuNP aggregates are present in the mixture. We have based our investigation on the hypothesis that the probability to detect a small object (protein-AuNP) is higher than larger ones (AuNP aggregates). Indeed, the nanoparticle capture rate (*f*) in a nanopore is mainly governed by diffusion. It can be defined as f=2πcDr, where *c* and *D* are the concentration and the diffusion coefficient of the NP, respectively, and *r* is the capture radius. Thus, assuming equilibrium where both free protein-AuNP and AuNP aggregates co-exist, we can expect that the free protein-AuNPs are detected without huge perturbations from the AuNP aggregates. The protein corona sensing with a solid-state nanopore constitutes an interesting improvement compared to the optical methods.

For our investigation, we chose a panel of five proteins presenting different structural properties. The BSA and peroxidase are classified in the CATH database [34,35] as mainly-α (composed by α-helix). The avidin and β-lactoglobulin are mainly-β (mainly composed by β-sheets). The glucose oxidase is classified as a mix-αβ (composed by both α-helix and β-sheets). We first investigate the impact of the protein on the Au-NP (diameter 10 nm) stability after NaCl addition. Then, we evaluate the volume of the protein corona using single nanopore technology.

## 2. Materials and Methods

### 2.1. Materials

Gold nanoparticles suspended in water with a nominal diameter of 10 nm (ref 752,584 lot MKBX1673V), albumin from bovine serum (BSA, A2153), avidin from egg white (A9275), glucose oxidase from Aspergillus niger (G7141), peroxidase from horseradish (P8250), β-lactoglobulin from bovine milk (L3908), sodium chloride (S753), sulfuric acid ACS reagent 95–98% (32051), hydrogen peroxide wt.30% (216763) were purchased from Sigma-Aldrich (Lyon, France). Silicon nitride grids (SiN) (20 nm thick and 50 × 50 µm windows) were purchased from Nanopore solution (Lisbon, Portugal). Ultra-pure water was produced from a Q-grad^®^-1 MilliQ system (Millipore, Lyon, France). mPEG 5 kDa silane (JKA3037-16) was purchased from Nanocs (New York, NY, USA).

### 2.2. Preparation of the Gold Nanoparticle-Protein Mixtures

The following procedure was used for all proteins (Peroxidase, BSA, avidin, glucose oxidase and β-lactoglobulin). The protein was directly added to the AuNP dispersed in water, with a concentration of 6 × 10^12^ particles mL^−1^. Before addition to the AuNP solution, the protein solution was filtered with a 0.22 µm filter. An aliquot of the protein was injected in the nanoparticle so as to maintain ratio at 20:1 throughout all the experiments. The final protein-nanoparticle solution was left for 2 h before use in order to let the proteins react with the gold nanoparticles.

### 2.3. Solid-State Nanopore Drilling and Functionalization

The SiNx membrane (thickness 30 nm) of a TEM grid was drilled by the electron beam (beam current 11 nA) of a transmission electron microscope (JEOL 2010F, Walter Uhl, Wetzlar, Germany) to obtain a nanopore with a diameter of 17 nm. The process to obtain the nanopore consisted of two steps. The membrane was first drilled with a 1 nm probe for 60 s. Afterwards, a 20 nm electron beam was employed for 120 s to illuminate and enlarge the previous hole until reaching the desired diameter. The resulting nanopore had a diameter of 17 nm. The functionalization with PEG silane was prepared as previously reported [24]. Briefly, the silicon surface was cleaned and activated in a piranha solution (H_2_SO_4_/H_2_O_2_ with a ratio 3:1 for 30 min at room temperature), rinsed with milliQ water, dried at 60 °C for 5 min to remove residual water droplets. Then the nanopore chip was incubated into 1.0 mg mL^−1^ PEG silane (5 kDa) in ethanol for 1 h, rinsed with ethanol, then with water and finally dried with nitrogen flow.

### 2.4. Dynamic Light Scattering Measurements

The diffusion coefficients of the gold nanoparticle-protein mixtures have been measured using Photon Cross-Correlation Spectroscopy (PCCS) (Nanophox Sympatec, Paris, France). The experiments were performed at 25 °C. The data from the DLS experiments were acquired for more than one hour, due to the low concentration of the protein-nanoparticle complex in the sample in order to replicate the same conditions as the nanopore experiments (3 × 10^9^ gold nanoparticles mL^−1^). The diffusion coefficients were obtained fitting the raw data with the Quickfit software. The experimental data were fitted with a Liverberg-Marquardt non-linear algorithm without the constraint box.

### 2.5. Gold Nanoparticle-Protein Complex Detection through SiN_x_ Nanopore

The single nanopore, once cleaned with piranha and functionalized with PEG, was placed in a Teflon cell containing 250 mM NaCl solution. Two Ag/AgCl electrodes were used to measure the current due to the presence of the ionic medium. One electrode was plugged to the positive end of the amplifier (*trans* chamber) and the other electrode connected to the ground (*cis* chamber). Initially, the gold nanoparticle-protein complex (nanoparticle concentration of 3 × 10^9^ particles mL^−1^, a protein/AuNP ratio 20:1) were injected in the cis chamber just before the transport experiment through the nanopore. A constant voltage (300 mV) was applied to generate an electric field between the *trans* and *cis* chamber and favor the translocation and thus, the detection of the protein-AuNp through the nanopore. The choice of this optimal voltage was determined by the number of events detected. At lower voltages we detected only few events, while at higher ones, we could have caused clogging of the sensor. The ionic current was recorded using a patch-clamp amplifier (EPC 800, HEKA electronics, Reutlingen, Germany) at a sampling frequency of 20 kHz. A 10 kHz filter was applied. The data acquisition was performed with a HEKA LIH 8 + 8 acquisition card using patch master software (HEKA electronics, Reutlingen, Germany). The event analysis was performed using the Matlab (Version 2016b, Matworks, Natick, MA, USA) code developed by Plesa et al. [36]. The event detection was carried out defining a threshold by multiplying a peak detection factor and the *rms* noise level calculated by the global standard deviation methods. In this work, the peak detection factor has been fixed at about 5. The SiN_x_ nanopores were cleaned with a water flow at the end of every experiment involving a specific protein-nanoparticle complex so to reuse it with another protein-gold nanoparticle complex.

## 3. Results and Discussion

### 3.1. Influence of Different Proteins on Nanoparticle Aggregation

**Figure 1:**
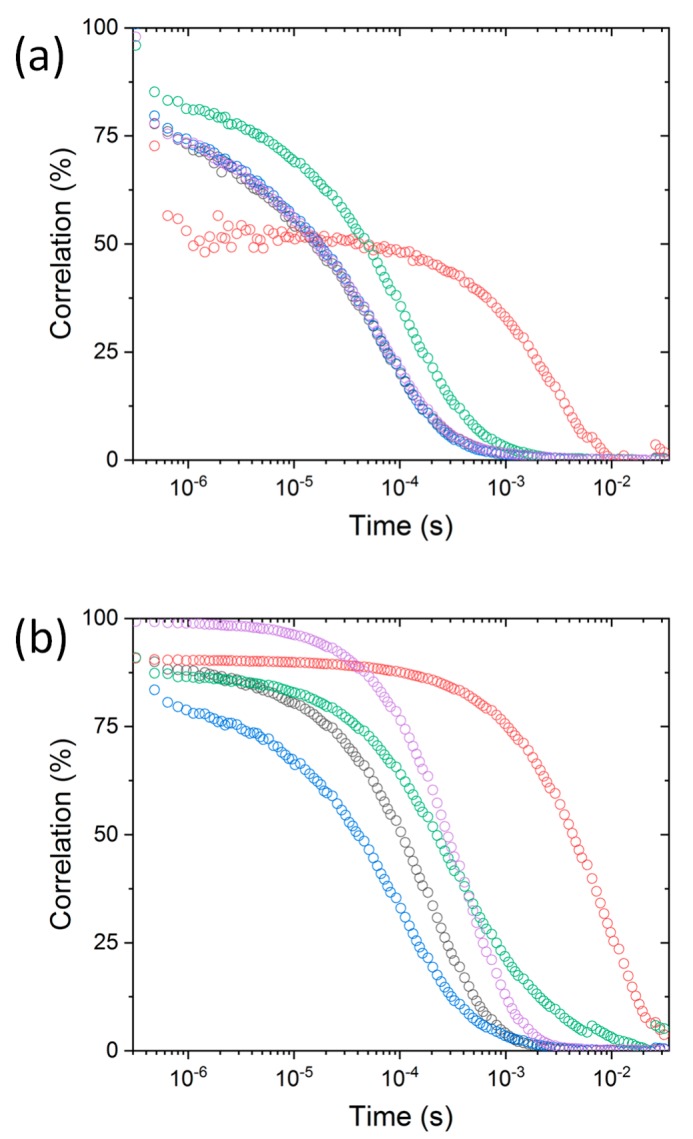
Dynamic light scattering curves from avidin-AuNP (red), β-lactoglobulin-AuNP (black), BSA-AuNP (blue), glucose oxidase-AuNP (green), peroxidase-AuNP (purple) at NaCl 10 mM (**a**) and 250 mM (**b**).

It is well known that the increase of NaCl concentration leads bare Au-NPs in solution to aggregate. On the other hand, proteins such as BSA can stabilize AuNP colloidal suspensions [37,38]. We investigated the effect of a protein panel with different structures (avidin, β-lactoglobulin, glucose oxidase, BSA and peroxidase) on the colloidal stability of the gold nanoparticles. We fixed the protein to nanoparticle ratio to 20:1 in accordance with our previous work on BSA [33]. This ratio was found as an optimal condition to prevent the aggregation of AuNP. The same ratio was used for the other protein-Au-NPs mixtures to have exactly the same conditions in all the experiments with DLS or with the solid-state nanopore. Even if all the proteins do not contribute to the corona formation, we were aware that free proteins are not detected by the nanopore functionalized with PEG, since their translocation is too fast. On the other hand, small aggregates can be easily distinguished from the nanoparticle-protein corona complex and big aggregates cannot pass through the 17 nm hole. In Figure 1, the DLS curves for the protein-AuNP for two NaCl concentrations (10 mM and 250 mM) are reported. It should be noted that the autocorrelation functions were collected at the same colloidal concentrations as those used in the nanopore experiments. This is necessary because both the protein and the AuNPs concentration can strongly influence the stability of the colloidal solution. The DLS results at low concentrations of protein and AuNP do not provide their exact size due to the low quality of the signal. However, it can offer a qualitative description of the initial state before and after salt addition.

At 10 mM NaCl, we observe that the correlation function is shifted toward a longer time for avidin and glucose oxidase. This means that these proteins are less efficient to prevent the nanoparticle aggregation than the other proteins (Figure 1a). Interestingly, with the increase of salt concentration (250 mM NaCl), the protein influence is also different. Indeed, only the BSA efficiently prevents nanoparticle aggregation. The shift of correlation function toward longer times follows this sequence: β-lactoblogulin, glucose oxidase, peroxidase and avidin.

### 3.2. Characterization of the Species by Single Nanopore Technique

**Figure 2:**
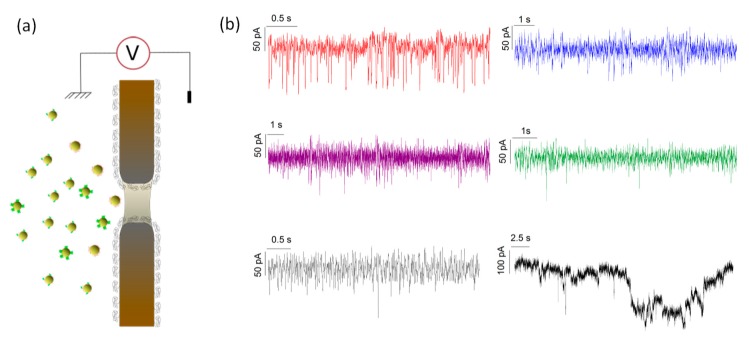
(**a**) Illustration of the nanopore sensing experiment (**b**) examples of current traces obtained from avidin-AuNP (red), BSA-AuNP (blue), peroxidase-AuNP (purple), glucose oxidase-AuNP (green), β-lactoglobulin-AuNP (black).

The second step was to study the composition of the protein-AuNP mixture with a method more sensitive to the small colloid than to the large one. In other words, we investigated the volume of the first layer of the protein corona around a single AuNP. To do so, we used single SiN_x_ nanopore for the detection of a single particle in solution. We performed different experiments involving the 10 nm gold nanoparticles mixed in solution with avidin, β-lactoglobulin, BSA, glucose oxidase, and peroxidase. In the first place, gold nanoparticles (*c* = 3 × 10^9^ particles mL^−1^) with BSA (ratio 20:1) were directly injected, in the *cis* chamber before the beginning of the transport experiment (Figure 2). The nanopore was functionalized by grafting PEG-silanes in order to improve its wettability, its lifetime [39,40] and prevent the protein unspecific adsorption [24]. The success of the nanopore functionalization was verified by the dependence of conductance vs. NaCl concentration as previously reported [24]. In addition, the PEG functionalization prevents the free protein/nanopore interactions. This limits the interferences with the protein-nanoparticle complex, because the free protein translocation is too fast to be detected at 20 kHz. We performed experiments at an NaCl concentration of 250 mM. At this salt concentration, the DLS experiment shows NP aggregates. However, big aggregates cannot pass through the 17 nm aperture of the nanochannel.

In Figure 2b, selected current traces recorded after the addition of protein-AuNP are reported. We can observe current blockades which can be assigned to the colloids passage through the nanopore. As mentioned before, because of the nanopore diameter (17 nm), the aggregates will clog it for a certain time, as shown Figure 2b (see black current trace). We can notice that the low diffusion coefficient and concentration of the aggregates make such events very rare and [41] they can be easily removed during data analysis.

**Figure 3:**
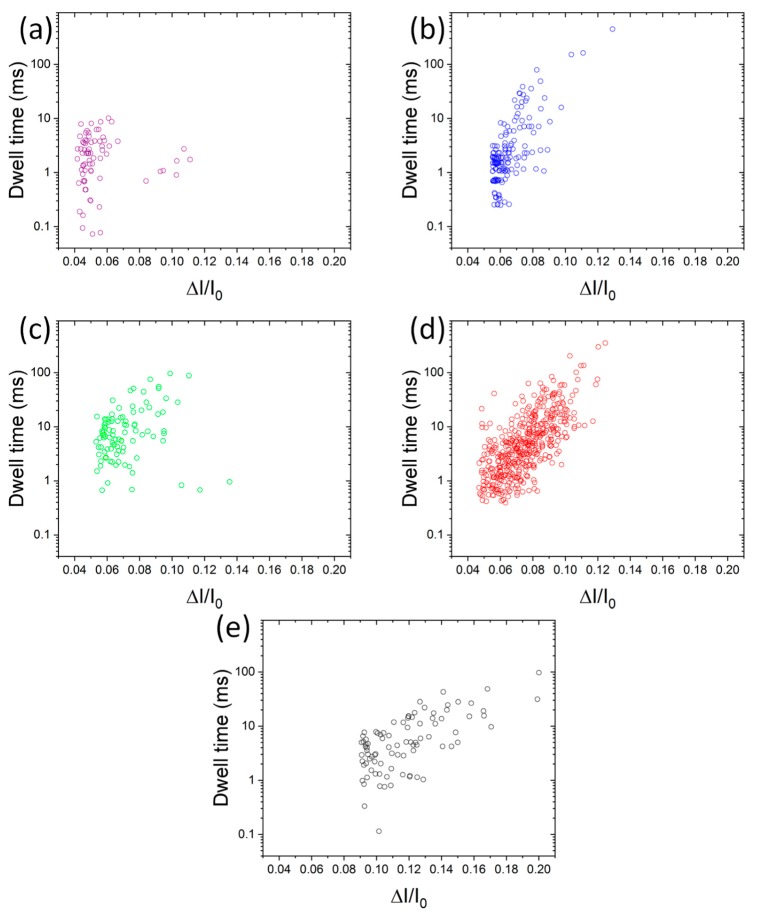
Events map of blockade parameters induced by peroxidase-AuNP ((**a**), purple), BSA-AuNP ((**b**), blue), glucose oxidase-AuNP ((**c**), green), avidin-AuNP ((**d**), red), β-lactoglobulin-AuNP ((**e**), black).

We focus our work on the analysis of the current blockades generated from the translocation across the nanopores. We analyzed the intensity of the relative current blockade Δ*I*/*I*_0_ and the dwell time Δ*t*. (Figure 3) At first look, we can see that it is possible to distinguish the different protein-AuNP samples. Indeed, the gold nanoparticles with their different protein layers are represented by discernable populations.

**Figure 4:**
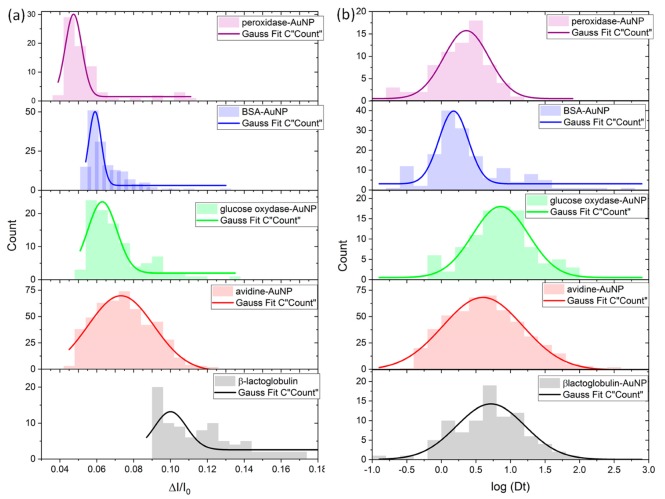
Distribution histograms of relative current blockade (**a**) and dwell time (**b**) obtained for peroxidase-AuNP (purple), BSA-AuNP (blue), glucose oxidase-AuNP (green), avidin-AuNP (red), β-lactoglobulin-AuNP (black).

To further investigate, the distribution histograms of both Δ*I*/*I*_0_ and the dwell time are reported for each protein-AuNp colloidal solution in Figure 4. The Δ*I*/*I*_0_ distributions were fitted by a single Gaussian function centered at 0.047, 0.059 0.062, 0.074 and 0.10 for peroxidase-AuNP, BSA-AuNP, glucose oxidase-AuNP, avidin-AuNP and β-lactoglobulin-AuNP, respectively. We can notice that the distribution for β-lactoglobulin-AuNP is widely spread. This could be interpreted by presence of the β-lactoglobulin dimers. We can notice that the mean amplitude of the relative current blockade follows the β-sheet content of the protein. If this trend is clear for the Δ*I*/*I*_0_, this is not the case for the dwell time distributions (Figure 4). Indeed, the dwell time distribution are centered to 1.82 ms 1.79 ms, 7.41 ms, 2.29 ms and 5.12 ms for peroxidase-AuNP, BSA-AuNP, glucose oxidase-AuNP, avidin-AuNP and β-lactoglobulin-AuNP, respectively. This is not surprising, since the dwell time involves the diffusion and the charge of colloids as well as their interaction with the surface [25]. This usually makes the dwell time less sensitive to the relative current blockade to discriminate the colloidal size. However, the BSA-AuNP and peroxidase-AuNP show shorter dwell time than the others, that is, avidin-AuNP, glucose oxidase-AuNP and β-lactoglobulin-AuNP.

To estimate the size of the objects passing through the nanopore, we used the Δ*I*/*I*_0_ distribution. There are several models in the literature to calculate the volume occupied by an object from the Δ*I*/*I*_0_. For instance, Yusko et al. proposed a model based on only the geometry to take into account the shape and volume of object [31,32]. This model is efficient to predict the current blockades caused by protein translocation. However, to take into account the nanopore surface charge, we use a variant of Yusco’s model that we recently proposed [28].

(1) $$\frac{\Delta I}{I_{0}}=\frac{4\gamma \Lambda }{\pi d_{p}^{2}\left(l_{p}+0.8d_{p}\right)}S\frac{r_{p}}{d_{M}}-\left(\frac{{µ}_{+}\sigma_{NP}S_{NP}-{µ}_{+}\sigma_{p}S_{p}}{\left({µ}_{+}+{µ}_{-}\right)c_{e}NV_{p}}\right)$$

where *r_p_*, *l_p_* γ is a form factor equal to 1.5 for a spherical geometry, and $S\frac{r_{p}}{d_{M}}$ is a correction factor as rpdM=11−0.8(Rhrp)3≈1, ${µ}_{+}$ and ${µ}_{-}$ are the mobility of Na^+^ and Cl^−^, $c_{e}$ is the concentration of NaCl, $S_{p}$ the surface of the nanopore, $V_{p}$ is the volume of the nanopore, Λ and $S_{NP}$ are the volume and the surface of the nanoparticle present inside the nanopore, respectively. In order to determine the mean volume of the protein-AuNP colloids, we use the center value of Δ*I*/*I*_0_ distribution histograms. Assuming a spherical geometry (Table 1), the corona volume was obtained after the evaluation of the protein/Au-NP complex (nominal nanoparticle diameter of 10 nm).

Following the increase of current blockades and the volume of the protein corona, outstanding information can be discussed. First, the trend does not agree with the DLS results. In other words, there is no correlation between the protein corona volume and the colloidal stability with the NaCl. The second piece of information is more interesting: The corona volume does not follow the protein volume but the β-sheet content. Indeed, the peroxidase and BSA are “soft” protein and mainly-α protein. Their volumes of the corona are the smallest. The glucose oxidase is classified as a mix-α/β protein. Its corona volume is a slightly larger than for the BSA. The biggest values for the corona volume are obtained for the two mainly-β proteins, avidin and β-lactoglobulin. From the results, one can speculate that structural modifications of peroxidase and BSA occur to minimize their internal energy on the AuNP surface [42,43], forming a thinner and more uniform layer. It can be explained by their “soft” nature that can be directly assigned to their secondary structure mainly composed by α-helices [44,45,46,47]. Conversely, the proteins mainly composed by β–sheets require more energy to modify their structures than α-proteins. Thus, it is plausible that their structures are less modified explaining the larger corona volume around the AuNP. We can also observe that the Δ*I*/*I*_0_ distributions are wider for the mainly-β proteins than for the mainly-α one. This can also be explained by the ability of the protein to modify its structure on the AuNP surface. Indeed, the soft protein will optimize its interaction with the surface and, thus, will spread on the AuNP surface. In this case, the coverage will be more homogeneous between the different particles than for the mainly-β proteins.

## 4. Conclusions

To sum up, our work aimed to investigate the formation of the protein corona under salt condition when both single protein-AuNP and aggregates co-exist in the solution. First, we found that the detected events coming from the translocation of single protein-AuNPs can be easily distinguished from aggregates, for which the signal can be easily removed by the data analysis. Moreover, we found that the protein corona size does not follow the size of the constituting protein but is a structural category. Basically, mainly-α proteins form a thinner corona than the mainly-β ones. These results lead us to consider the protein structural category, information easily accessible from the CATH database, as an essential parameter for the study of the protein corona. We expect that this work will open a new avenue toward a predictive model for the protein adsorption on the nanoparticle and consequently the protein corona formation. This will be a new step for numerous teragnostic and sensing applications as well as for the understanding of the nanoparticle impact on health, food and the environment. In these domains, proteins are always in the surroundings of the nanoparticle and we cannot neglect their presence and role.

## Figures and Tables

**Table 1:** Results obtained from the relative current blockade. The error is the full width at half maximum (the corona volume is calculated from colloid volume—AuNP volume).

	Δ*I*/*I*_0_	Colloids Volume (nm^3^)	Corona Volume (nm^3^)	Hydrodynamic Radius of Protein (nm)
peroxidase-AuNP	0.047 ± 0.011	1433 ± 73	909	3
BSA-AuNP	0.059 ± 0.008	1512 ± 53	989	3.5
glucose oxidase-AuNP	0.062 ± 0.0062	1532 ± 409	1008	4.3
avidin-AuNP	0.074 ± 0.041	1611 ± 277	1087	3.9
β-lactoglobulin-AuNP	0.100 ± 0.020	1783 ± 132	1259	2.9
